# Tumor grafts derived from patients with head and neck squamous carcinoma authentically maintain the molecular and histologic characteristics of human cancers

**DOI:** 10.1186/1479-5876-11-198

**Published:** 2013-08-27

**Authors:** Shaohua Peng, Chad J Creighton, Yiqun Zhang, Banibrata Sen, Tuhina Mazumdar, Jeffery N Myers, Adrian Woolfson, Matthew V Lorenzi, Diana Bell, Michelle D Williams, Faye M Johnson

**Affiliations:** 1 Departments of Thoracic/Head and Neck Medical Oncology, Unit 432, The University of Texas MD Anderson Cancer Center, 1515 Holcombe Boulevard, Houston 77030-4009, TX, USA; 2 Dan L. Duncan Cancer Center, Baylor College of Medicine, Houston, TX, USA; 3 Head and Neck Surgery, The University of Texas MD Anderson Cancer Center, Houston 77030-4009, TX, USA; 4 Pathology, The University of Texas MD Anderson Cancer Center, Houston 77030-4009, TX, USA; 5 Discovery Oncology, Bristol-Myers Squibb Company, Princeton, NJ, USA; 6 Department of Medicine, Baylor College of Medicine, Houston, TX, USA; 7 Bioinformatics and Computational Biology, The University of Texas MD Anderson Cancer Center, Houston, TX, USA; 8 The University of Texas Graduate School of Biomedical Sciences at Houston, Houston, TX, USA

**Keywords:** Patient-derived xenograft, Translational animal models, Gene expression, Head and neck cancer

## Abstract

**Background:**

The patient-derived xenograft (PDX) model is likely to reflect human tumor biology more accurately than cultured cell lines because human tumors are implanted directly into animals; maintained in an *in vivo*, three-dimensional environment; and never cultured on plastic. PDX models of head and neck squamous cell carcinoma (HNSCC) have been developed previously but were not well characterized at the molecular level. HNSCC is a deadly and disfiguring disease for which better systemic therapy is desperately needed. The development of new therapies and the understanding of HNSCC biology both depend upon clinically relevant animal models. We developed and characterized the patient-derived xenograft (PDX) model because it is likely to recapitulate human tumor biology.

**Methods:**

We transplanted 30 primary tumors directly into mice. The histology and stromal components were analyzed by immunohistochemistry. Gene expression analysis was conducted on patient tumors and on PDXs and cell lines derived from one PDX and from independent, human tumors.

**Results:**

Five of 30 (17%) transplanted tumors could be serially passaged. Engraftment was more frequent among HNSCC with poor differentiation and nodal disease. The tumors maintained the histologic characteristics of the parent tumor, although human stromal components were lost upon engraftment. The degree of difference in gene expression between the PDX and its parent tumor varied widely but was stable up to the tenth generation in one PDX. For genes whose expression differed between parent tumors and cell lines in culture, the PDX expression pattern was very similar to that of the parent tumor. There were also significant expression differences between the human tumors that subsequently grew in mice and those that did not, suggesting that this model enriches for cancers with distinct biological features. The PDX model was used successfully to test targeted drugs in vivo.

**Conclusion:**

The PDX model for HNSCC is feasible, recapitulates the histology of the original tumor, and generates stable gene expression patterns. Gene expression patterns and histology suggested that the PDX more closely recapitulated the parental tumor than did cells in culture. Thus, the PDX is a robust model in which to evaluate tumor biology and novel therapeutics.

## Background

Approximately 52,610 new cases of head and neck cancer are diagnosed in the United States each year and worldwide annual incidence is estimated at 644,000 [1,2]. While locoregional control in advanced head and neck squamous cell carcinoma (HNSCC) has improved, recurrence is still common. Even when curative therapy is available, HNSCC is often a disabling and disfiguring cancer that can have a profound impact on important functions such as eating, speaking, sight, and hearing. These insults are compounded by distortions in facial appearance. To advance therapy for HNSCC, better laboratory models are needed to study HNSCC biology, systemic therapy, and radiotherapy.

There are several existing animal models for head and neck cancers [3,4]. No one model is ideal, and each has its advantages and disadvantages. These include chemical-induced cancer models; syngeneic murine cancer cells injected back into immunocompetent mice from the same strain; transgenic mice expressing mutant *KRAS*[5,6] or activated *AKT* with *p53* loss [7]; and xenograft models in which human HNSCC cell lines, grown on plastic in tissue culture, are injected into immunocompromised mice, either subcutaneously or into an orthotopic site such as the tongue or the floor of the mouth [8]. The orthotopic xenograft model is appealing because it uses human cancer cells in an appropriate anatomical site, is reliable, and recapitulates, to some extent, human tumor behavior. However, a major disadvantage of models that rely on cells grown on plastic is that these HNSCC cell lines have gene expression profiles that are markedly different from HNSCC tumors from patients [9].

An increasingly promising xenograft model, the patient-derived xenograft (PDX), is developed by surgically implanting tumor tissue directly from a patient into an immunocompromised mouse. The resulting heterotransplanted tumors maintain the histologic characteristics of the primary tumor [10-13], and the pattern of response to chemotherapy resembles those observed in the clinic [14-17]. PDXs of non-small cell lung cancer (NSCLC) were shown to maintain the gene expression patterns of the original tumor [18]. Furthermore, the PDX model utilizes tumors from several individuals, suggesting that this approach could serve as a better surrogate for therapeutic studies in human HNSCC.

The purpose of our study is to generate HNSCC PDXs and characterize how well the model recapitulates human disease. Our hypothesis is that human HNSCC tumor tissue transplanted directly into nude mice maintains the molecular and histologic features of the original tumors. An additional unanswered question is the origin of the stromal components observed in PDX models. We transplanted 30 human HNSCC tumors directly into mice and serially transplanted those that engrafted. The histology was compared in the parental and PDX tumors. The origin of the stromal components was analyzed using mouse- and human- specific antibodies. Gene expression analysis was conducted on patient tumors and on PDXs and cell lines. This is the first published study of an HNSCC PDX model that has been characterized at the molecular level.

## Methods

### HNSCC patient tumor engraftment into mice

Residual tumor was taken at the time of surgery from 26 previously untreated patients undergoing definitive surgery and 4 patients undergoing surgical salvage for HNSCC. Upon arrival in the pathology suite, these tissues were transported immediately to the animal facility in sterile RPMI medium. For samples measuring <0.5 cm^3^, the entire sample was implanted into a single nude mouse. For samples measuring >0.5 cm^3^, the original patients’ tumors (F0 generation) were divided; part of the tissue was implanted in a mouse, and the remaining portion was snap-frozen in liquid nitrogen or stored in RNAlater (Life Technologies, Carlsbad, CA). The tumor tissue used for implantation was minced into 2-mm^3^ pieces, which were implanted subcutaneously into the flanks of anesthetized 6-week-old Nu/Nu female mice that were bred onsite at MD Anderson. When the resulting tumors grew to 1 cm^3^, each tumor (F1 generation) was resected and divided as for the primary tumor and passaged into 5 mice (F2 generation). The process was repeated to produce subsequent generations. The tumors and the derived PDXs were named human oral squamous carcinoma HOSC 1-30; all PDX models maintained the same HOSC number as the parent tumor from which they were derived. All animal studies were performed in accordance with the policies of the Institutional Animal Care and Use Committee and were approved by the Institutional Review Board of The University of Texas MD Anderson Cancer Center.

### Cell culture and lines established from xenografts

At the time of xenograft passage, remaining viable tumor tissue was reduced to 1- to 2-mm^3^ fragments, which were transferred to Dulbecco modified Eagle medium containing 10% FBS, 100 U/ml penicillin, and 100 μg/ml streptomycin (Sigma, St. Louis, MO) and incubated at 37°C in an atmosphere containing 5% CO_2_. The medium was renewed twice weekly once the cells had become attached. For 3D cell culture, the Bio-Assembler 3D culture system (n3D Biosciences, Inc., Houston, TX) was adopted according to the manufacturer’s instructions [19]. Established HNSCC cell lines (Tu167 and Osc19) were maintained as previously described [20].

### Histologic characterization

Tumor tissues from parent tumors and PDXs were formalin fixed, paraffin-embedded and stained with hematoxylin and eosin. The tumors were examined under light microscopy by a head and neck pathologist (M.D.W.). Tumors were evaluated for the degree of differentiation (formation of keratin, cytologic features, and growth pattern), presence of perineural invasion, desmoplastic stroma and extent of inflammation. The patient’s surgical resection was also microscopically evaluated for lymph node metastases and presence or absence of extranodal extension.

### Immunohistochemistry

Immunohistochemical (IHC) analysis was performed as described previously [21,22]. Briefly, 5-μm, paraffin-embedded tumor sections were deparaffinized, rehydrated, and subjected to antigen retrieval in sodium citrate buffer (pH 6.0). Slides were quenched in 3% H_2_O_2_ for 15 min, rinsed in PBS, blocked in avidin for 10 min, rinsed in PBS, blocked in biotin for 10 min, and washed and blocked in whole serum for 15 min. Slides were incubated with primary antibody (human specific anti-vimentin [Biocare Medical, Concord, CA] or human/mouse-specific anti-vimentin [Thermo Scientific, Hanover Park, IL]) or human-specific keratin 5/6 (DAKO, Glostrup, Denmark) or PCNA (Biocare Medical) for 30 min, rinsed in PBS, incubated in biotinylated anti-mouse IgG, rinsed in PBS, then incubated in streptavidin-horseradish peroxidase. The chromagen 3-amino-9-ethylcarbozole (AEC) or 3,3′-diaminobenzidine (DAB) were used to detect antigen. Slides were counterstained with hematoxylin. Nuclear PCNA expression was quantified in 20 fields per sample using a 3-value intensity: 0, none; low (weak to moderate); and high (strong).

### Gene expression array

RNA was extracted from parent tumors, xenografts, and both PDX–derived and established HNSCC cell lines using an RNAeasy mini kit (Qiagen, Valencia, CA). The integrity of the RNA from each sample was measured by using the RNA 6000 Nano LabChip and a 2100 Bioanalyzer (Agilent Technologies, Palo Alto, CA). The quality and concentration of RNA was assessed on an ND-1000 spectrophotometer (NanoDrop, Wilmington, DE). The Affymetrix (Santa Clara, CA) GeneChip U133 Plus 2.0 was used without RNA amplification. Hybridization, washing, staining, and scanning were performed by Asuragen, Inc. (Austin, TX) as previously described [23]. All samples were run in a single batch to avoid batch effects.

Gene array data were quantile normalized; differential gene expression was assessed by two-sided *t*-test and is expressed as fold-change (using log-transformed data). Hierarchical clustering trees were generated by Eisen Cluster software [24]. Heat maps were generated by JavaTreeView [25]. Array data have been deposited in the Gene Expression Omnibus (GEO; accession GSE45153).

### Drug treatment of mice

HOSC1-F7 tumors from 8 mice were passaged into 40 nude mice using the techniques described above. Once the tumors reached approximately 0.5 cm^3^ the mice were stratified for tumor size by TM into 4 groups which were then randomly assigned one of 4 treatment regimens by a researcher blinded to the stratification (SP). Dasatinib (20 mg/kg), BMS911543 (10 mg/kg), both, or vehicle was administered by oral gavage daily for 16 days. Dasatinib was purchased from the clinical pharmacy and BMS911543 was provided by the Bristol-Myers Squibb Company. Mice were killed 2 hours following the last drug dose, tumors were dissected, and the mice examined for distant metastases. The tumors were fixed and subjected to histological and IHC analysis as described previously [26].

### Real time PCR

We extracted mRNA from HOSC1-F0 and HOSC1-F3 tumors using the Qiagen RNeasy minikit; synthesized cDNA using the Promega RT-PCR system; applied primers designed using Primer-Blast; and performed RT-PCR using the SYBR Green chemistry as we previously described [27]. Expression of the L32 gene was used as an internal control.

## Results

### Generation of patient-derived HNSCC xenografts

**Figure 1:**
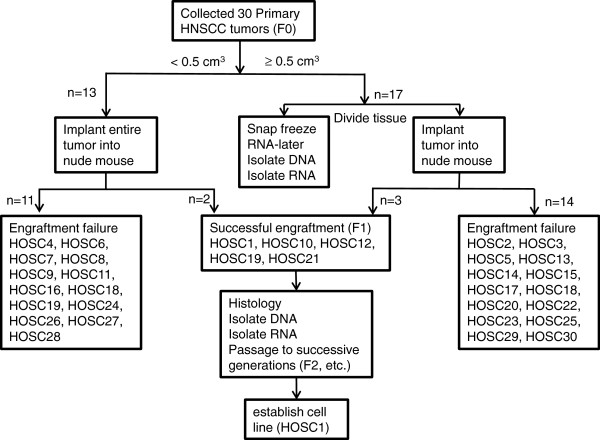
Flow sheet for the development of patient-derived xenografts and cell lines from parent head and neck cancer tissue specimens.

We received 30 residual tumor samples from surgically resected specimens. Thirteen of the tissue samples were small (<0.5 cm^3^) and were implanted in their entirety, whereas 17 larger tumors (>0.5 cm^3^) were divided to allow RNA/DNA collection (Figure 1). The tumors were predominantly oral squamous cancer, because these are most likely to undergo primary surgical resection. Five of the implanted tumors engrafted, for an overall engraftment rate of 17%. Selected patient characteristics are included in Additional file 1: Table S1.

### Poorly differentiated tumors with positive nodes were more likely to engraft

Engraftment was more common among poorly differentiated and node-positive tumors (differences not significant by chi-squared test), but was not associated with patient clinical outcome (recurrence) or T stage (Table 1). Of the 8 poorly differentiated tumors, 3 engrafted (38%); 2 of the 19 that were moderately differentiated engrafted (9%); and none of the 3 that were well differentiated engrafted. Of the 13 N0 tumors, 1 engrafted (8%), while 4 of the 13 N + tumors engrafted (31%). None of the 4 tumors derived from recurrent tumors engrafted. Gene expression analysis of tumors that engrafted and those that did not revealed 992 probes whose expression was distinctly different between the two groups (P < 0.01, fold-change >1.4) and that reflected diverse functions (Additional file 2: Figure S1 and Additional files 3, 4).

**Table 1:** Clinical and pathological characteristics of transplanted tumors

		**Total**	**Engrafted**	**Percent engrafted**
Differentiation	Poor	8	3	38
	Moderate	19	2	11
	Well	3	0	0
Extracapsular	Present	8	2	25
Extension	Absent	12	3	25
	No nodes	10	0	0
Perineural	Present	11	3	27
Invasion	Absent	19	2	11
T Stage	T1/T2	8	2	25
	T3/T4	18	3	17
	Recurrent	4	0	0
N Stage	N0	13	1	8
	N1-3	13	4	31
	Recurrent	4	0	0
Recurrence	Present	13	2	15
	Absent	17	3	18

### Patient-derived xenografts maintained the histologic characteristics of the tumor from which they were derived

**Figure 2:**
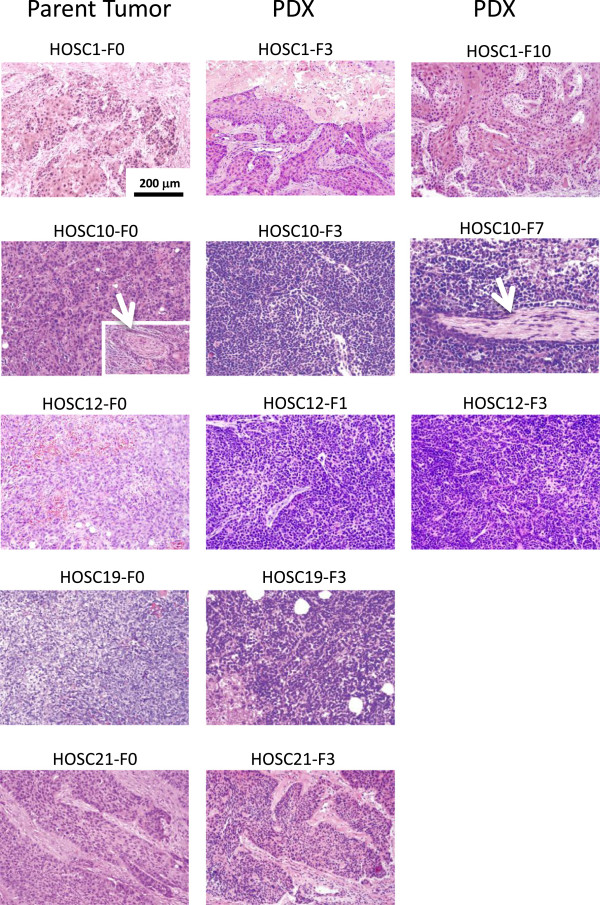
**PDXs maintained the histologic characteristics of the parent tumors from which they were derived.** Hematoxylin & eosin staining (200 x) of patients’ original tumor (F0) and PDX tumors demonstrated similar histologic characteristics, but with more homogeneity. Perineural invasion (white arrow) matched that of the parent tumor (HOSC10).

Patient-derived xenografts and the original human tumors were examined for histology, degree of differentiation, and presence of inflammation, perineural invasion, and necrosis (Additional file 1: Table S2). In all cases, the histology (invasive squamous carcinoma) and degree of differentiation of the PDX matched that of the parent tumor (Figure 2). There was a general trend for the PDX tumors to lose stroma and become more solid and homogeneous in successive generations. Mouse nerves were present for evaluation in 2 PDX tumors (HOSC10 and HOSC12), allowing evaluation for perineural invasion, which matched that of the parent tumor.

### Patient-derived xenografts lost human stromal components

**Figure 3:**
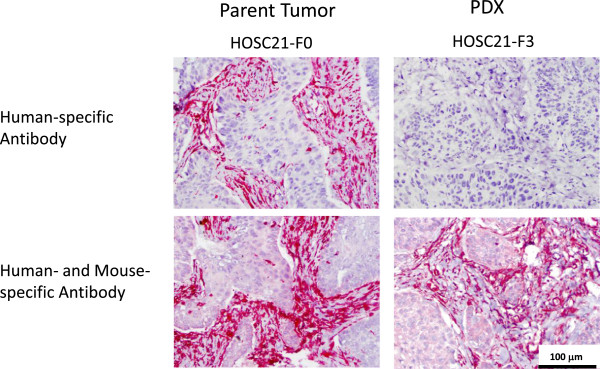
**PDX tumors contained exclusively murine stromal components.** Representative IHC is shown. Parental human tumors (F0) and PDXs (F3) were stained with anti-vimentin antibodies that are specific for human vimentin (top row) or both human and mouse vimentin (bottom row).

To determine whether the stroma observed in PDX tumors was of mouse or human origin, we utilized two anti-vimentin antibodies--one specific to human vimentin and a second that binds both human and mouse vimentin. Vimentin is a mesenchymal marker that stains fibroblasts and a fraction of squamous tumors that have undergone epithelial to mesenchymal transition [28]. No stromal staining for human vimentin was observed, however stroma did stain with the antibody which recognized both human and mouse (Figure 3, Additional file 1: Table S3, and Additional file 5: Figure S2). Only 2 of the PDXs had significant mouse stroma present; one had moderate mouse stroma, and the other 2 had only sparse mouse vessel staining. As expected, the human tumor cells in some of the PDX models stained weakly with vimentin. As a control we stained the PDX tumors for a human specific epithelial marker (keratin 5/6). As expected, there was no stromal staining. The tumor cell staining intensity varied widely between models (Additional file 6: Figure S3).

The growth rate of the 5 PDX models varied from model to model and the tumors tended to grow more quickly in subsequent generations (Additional file 7: Figure S4). The acceleration in growth rate is consistent with our finding that the cancer cell content of the tumors increased over time.

### Gene expression patterns of patient-derived xenografts and parent tumors were distinct from those of human cancer cells in culture

**Figure 4:**
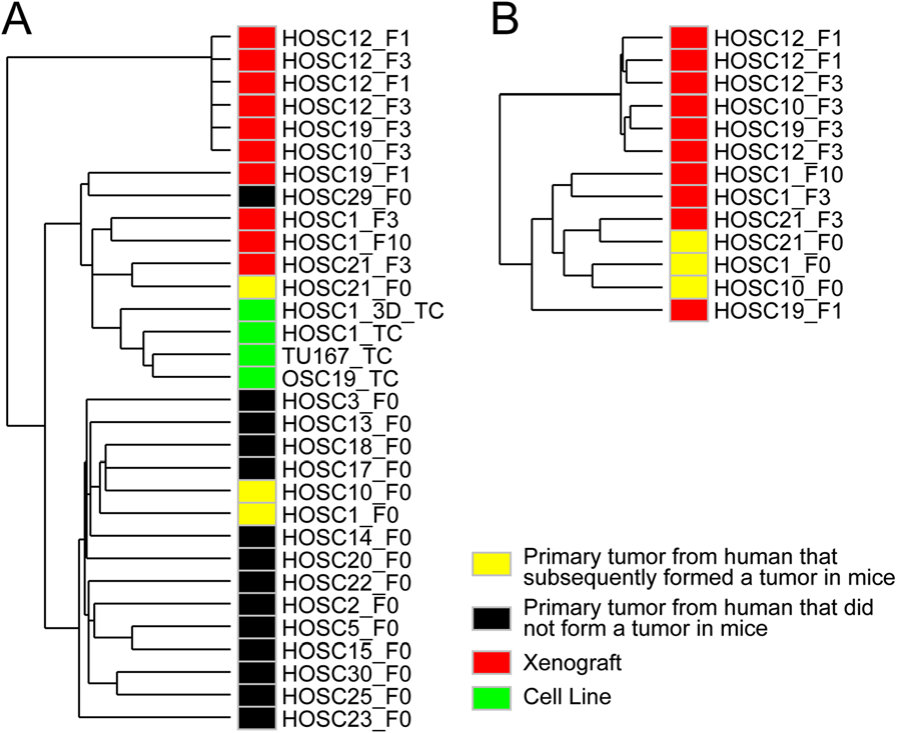
**Gene expression patterns of patient-derived xenografts, parent tumors, and human cancer cells in culture were distinct.** This similarity tree of samples is based on unsupervised clustering of gene expression in parental tumors, PDX, and cells in culture with all samples **(A)** or excluding cell lines and tumors that failed to engraft **(B)** (using expression values for all 54,000 gene transcript probes represented in the dataset). Samples that appear on proximate branches of the tree are more similar to each other in terms of their global expression patterns.

**Figure 5:**
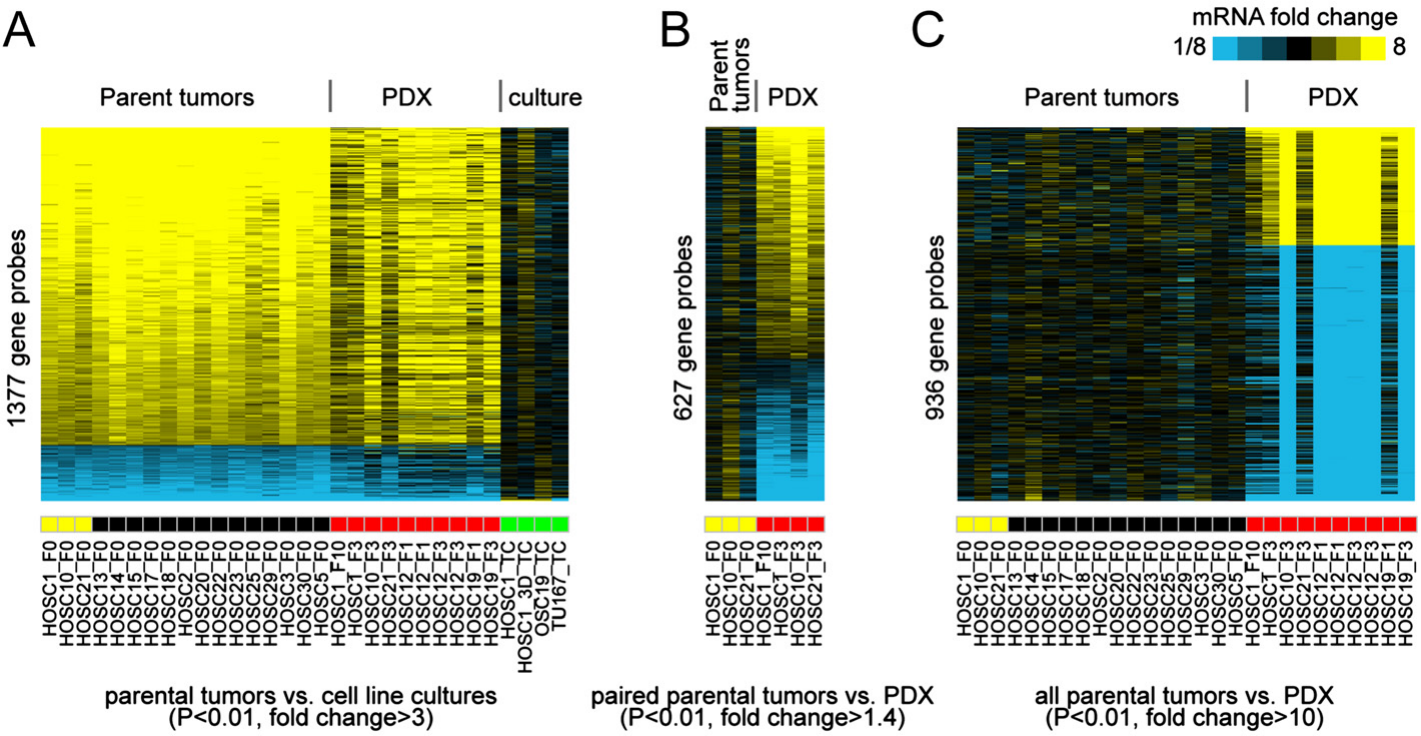
**Gene expression patterns of patient-derived xenografts and parent tumors were distinct from that of human cancer cells in culture. (A)** Analysis of gene expression data from parental tumors and human HNSCC cancer cells in culture revealed 1377 probes that were distinct (P < 0.01, unpaired *t*-test, fold-change >3 in either direction). **(B)** Comparison of 3 parental tumors to the paired PDXs revealed 627 probes that were distinct (P < 0.01, paired *t*-test, fold-change >1.4). **(C)** Comparison of all parental tumors to all PDXs revealed 936 probes that were distinct (P < 0.01, unpaired *t*-test, fold-change >10). Specific gene ontogeny groups are listed in Additional files 3, 4. For each heat map, genes are centered on a given reference group to show relative expression differences.

Many HNSCC mouse models utilize cancer cells grown in culture, but HNSCC cell lines have gene expression profiles that are markedly different from those of patient tumors [9]. We performed unsupervised clustering of gene expression (on the basis of 54,000 gene transcript probes represented in the dataset) from all the human parent tumors, PDXs, a cell line derived from HOSC1 PDX, HOSC1 grown under 3D conditions, and 2 established HNSCC cell lines (Figure 4). As expected, all the cell lines clustered tightly together and were distinct from the tumor samples. Likewise, we found 1377 gene probes that were different between HNSCC parent tumors and HNSCC human cell lines in culture (P < 0.01, fold-change >3). In contrast, the PDX expression pattern for these 1377 probes was quite similar to that of the human parent tumors (Figure 5A).

The majority of parent tumors clustered separately from the PDXs. Of the 3 matched pairs, one (HOSC21) clustered tightly with its xenograft, whereas the other 2 (HOSC1 and HOSC10) did not. No clinical or pathologic characteristics explained the better maintenance of gene expression in HOSC21 than in the other PDXs. The tight clustering of 2 generations of HOSC12 (F1 and F3) and HOSC1 (F3 and F10) demonstrated that gene expression was stable once the tumors were established in mice, although HOSC19 F1 and F3 did not cluster together.

Examining specific genes, we found 627 probes that were different between the PDX tumors and the parent tumors when only the 3 paired samples were analyzed (P < 0.01, fold-change >1.4) (Figure 5B). When all samples were analyzed, there were 936 distinct probes (Figure 5C). For genes with known functions, Additional files 8 and 9 list the gene ontogeny terms for genes with distinct expression. These include genes involved in cell adhesion and immune response. We chose 4 genes with distinct expression patterns between the parent tumor and the PDX model and confirmed expression differences using qPCR (Additional file 10: Figure S5).

### The PDX model of HNSCC can be used to test novel therapeutics

**Figure 6:**
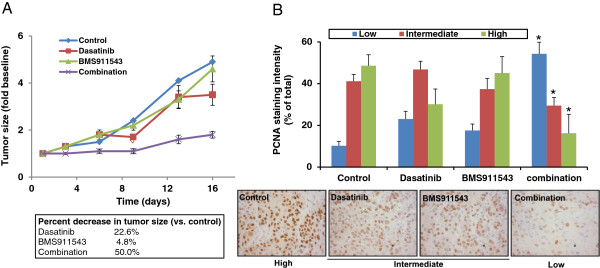
**The combination of Src and JAK inhibition decreases in vivo proliferation compared to single agents.** Nude mice bearing HOSC1 PDX tumors were treated with Dasatinib (20 mg/kg), BMS911543 (10 mg/kg), both, or vehicle for 16 days as indicated. **(A)** Tumor size was measured twice a week. **(B)** Tumors were stained for PCNA at the end of treatment and nuclear staining intensity scored and graphed. Representative IHC fields are shown. *p < 0.05 compared to control (vehicle treated).

Based on our previously published work, we hypothesized that JAK and Src inhibitors would have synergistic anti-tumor effects in vivo [26]. Mice bearing HOSC1 PDX tumors were treated with the Src inhibitor dasatinib, the JAK2 inhibitor BMS911543, both drugs, or vehicle control. Tumors were measured twice weekly and stained for PCNA to measure proliferation at the end of treatment. By day 16, growth of the tumors in the combination group had been inhibited by 63% (Figure 6A). Likewise, there was a significant decrease in PCNA staining intensity consistent with decreased proliferation (Figure 6B). Specifically, the percent of cells staining with low, intermediate, and high intensity was 10, 41, and 49% in the control tissues and 54, 29, and 16% in the tissues from mice treated with the combination therapy respectively.

## Discussion

In this study we transplanted 30 human HNSCC tumors directly into mice and serially transplanted those that engrafted. The overall engraftment rate was 17% with poorly differentiated and node-positive tumors more likely to engraft. There were significant expression differences between the human tumors that subsequently grew in mice and those that did not. The tumors maintained the histologic characteristics of the parent tumor, but were more homogeneous in successive generations. Human stromal components were lost upon engraftment. Gene expression between the PDXs and parent tumors were distinct, but stable in successive generations. The PDX expression pattern for gene probes that were different between parent tumors and human cell lines was quite similar to that of the human parent tumors.

Several PDX models have been developed and characterized, with several common themes emerging. In nearly all cases the histology of the original parent tumor was similar to that of the matched PDX [10-13]. This similarity persisted up to at least passage 12 [15]. In our study, tumors from passage 10 were histologically similar to parent tumors, with a loss of stroma over subsequent generations. Genetic abnormalities in the parent tumor such as specific mutations and gene copy number generally have been maintained in xenografts. In a study of colon cancer xenografts, there was concordance between gene copy number and mutation (*NRAS, KRAS, BRAF, PIK3CA*) in first- and second-generation xenografts and in the parent tumor [15]. Lung cancer models demonstrated similar karyotype and gene copy number as parent tumors by array comparative genomic hybridization (aCGH) [10].

The engraftment rate has varied greatly in different models, and rates ranging from 11% to 90% have been reported [11,29]. The highest engraftment rate was attributed to rigorous selection of viable cancer tissue and the relatively greater vascularity of the renal site of engraftment than of the usual subcutaneous site [16], although a subcutaneous colon cancer model had a similar engraftment rate of 87% [15]. Engraftment correlated with less tumor differentiation [11,30], advanced stage [13], and poor donor prognosis [12,29,31,32] in most, but not all, studies [11,33]. In the best characterized HNSCC PDX model before ours, the engraftment rate was 11% overall. In that model, poorly differentiated tumors, larger tumors, recurrent tumors, and tumors from lymph node metastases were more likely to form xenografts [30,33]. Additional PDX models of HNSCC have been developed but have not been well characterized at the molecular level and most also demonstrated low engraftment rates [34-40]. In our study, tumors that were poorly differentiated and node positive were significantly more likely to engraft.

Factors other than differentiation and metastasis to lymph nodes may also influence engraftment rates. Tumors derived from metastatic sites have generally been more likely to engraft than those from primary sites [15,30], but not always [29]. Surprisingly, cold ischemic time did not influence engraftment [15]. Characteristics that predicted engraftment in NSCLC included squamous histology, poor differentiation, larger tumor size, and *KRAS* mutation [12]. Likewise, in pancreatic cancer the tumors that grew in mice were more likely to have *SMAD4* mutations and an independently developed metastatic gene signature [31,41]. Efferth *et al*. examined 20 proteins in NSCLCs that were transplanted into mice and found that increased expression of JUN, FOS, cyclin D, and CDK4 predicted successful engraftment [42]. Our results show that tumors that engrafted had a gene expression profile that was distinct from the profile of those that did not engraft.

Few studies have rigorously examined stroma in PDX models. One of the most comprehensive characterizations of the PDX model showed that, after the first passage, tumor cells were enriched in the xenografts compared to the parent tumors, but the ratio of stroma:tumor was constant in subsequent passages. Human stroma was lost in the early passages [15]. In a breast cancer model, human stroma was replaced by mouse stroma after engraftment and tumor cells were enriched in the xenografts [29]. When NSCLC tumors were engrafted into NOD-scid IL2γ^null^ (NSG) mice, tumor-derived human leukocytes were maintained for up to 9 weeks in the first generation [43]. Similarly, we found that in our HNSCC PDX models, the human stroma was replaced by mouse stroma that was progressively less abundant in subsequent passages.

In the subcutaneous PDX models, metastases have not been described. However, nodal and distant (lung and liver) metastases have developed from orthotopic injection, in patterns consistent with clinical observations in colorectal cancer patients [15]. In a breast cancer model, both local nodal and distant metastases were found at rates that varied from 38% to 100% [29].

Several studies have compared gene expression in PDXs with that in the original parent tumors in lung, breast, and colon cancers. These studies show mixed results which are similar to ours; we found that the expression patterns of the PDX and parent tumors were distinct in 2 cases, but one parent tumor and PDX pair did cluster very tightly together. Unsupervised hierarchical clustering of 17 human NSCLC tumors and their matched heterotransplants revealed that 9 of the 17 animal tumors clustered with the primary tumors from which they were derived; 5 of the 8 primary tumors that did not cluster with their matched heterotransplants contained less than 10% tumor tissue [18]. In a breast cancer model, using expression of a set of 1291 intrinsic genes and unsupervised clustering, all xenografts and parent tumor pairs clustered together [29]. Unsupervised clustering of about 40 patient tumors with matched early- and late-stage xenografts separated parent tumors from xenografts, but there were no major differences between early- and late-stage xenografts. Ontogeny analysis of genes that were differentially expressed between tumors and xenografts revealed genes encoding for extracellular matrix components and immune modulators consistent with the loss of human stroma in the xenografts [15]. Gene expression analysis in breast cancer xenografts demonstrated that the majority of probes were not differentially expressed between the parent tumor and its xenograft. In this study, gene ontology analysis demonstrated that the genes that were differentially expressed corresponded to the stromal compartment [17]. Likewise, an independent group identified stromal and extracellular matrix genes as differentially expressed in xenografts and parent tumors [44].

Gene expression was analyzed in 3 small cell lung cancer (SCLC) biopsy specimens, primary xenografts derived from the tumors, cell lines derived from the xenografts, and those same lines reimplanted into mice. When compared to normal lung, the tumors, xenografts, and cell lines had an expression pattern that was specific for SCLC. However, the gene expression patterns changed when the cancer cells were grown on plastic, and this expression pattern did not fully recover when the lines were subsequently re-injected *in vivo* (i.e., secondary xenografts). The expression patterns of the primary xenografts more closely resembled those of the parental tumors than those of the cell lines or the secondary xenografts [45]. Likewise, in our model, the genes that were expressed differently in cell lines grown in plastic vs. parental tumors were maintained in PDX tumors, suggesting that the PDX model more closely reflects the biology of the parental tumor than do cell lines.

The ultimate goal of developing a PDX model is to recapitulate tumor biology for the development of effective cancer therapies. When xenografts are treated with therapeutic agents used in patients with that tumor, similar response rates are usually observed, suggesting that the PDX model is superior to cell culture models *in vitro* or *in vivo* for drug development. When mice bearing colorectal cancer xenografts were treated with cetuximab, the response rates mirrored those seen in clinical trials of cetuximab, and tumors bearing *KRAS* mutations were resistant to cetuximab, as are most *KRAS*-mutant colon cancers in human patients [14]. Similar results were demonstrated in a separate group of xenografts derived from colorectal cancer in which irinotecan and cetuximab as single agents had response rates in the population of xenografts that were similar to those found in human populations [15]. When mice with NSCLC xenografts were treated with chemotherapy regimens that are commonly administered to NSCLC patients, response rates were similar to those in patients [16]. Fifteen colorectal cancer xenografts responded to cytotoxic chemotherapy agents that are active in colon cancer in human patients [17].

Data linking drug sensitivity in specific patients to their paired xenografts are sparse. In a pancreatic cancer model, there was a positive correlation between responses of the xenografts to gemcitabine and time to progression for the corresponding 7 patients who were treated with gemcitabine [31]. The most comprehensive and only prospective published study established xenografts from 14 patients with advanced metastatic disease and screened the tumors for sensitivity to 63 drugs. Two of the patients’ xenografts were not sensitive to any drugs tested, but the other 12 patients underwent therapy based on the xenograft data. In 9 of the 12 patients, the cancer responded as predicted by this screening, while the two cancers whose xenografts were resistant did not respond to standard therapy [46]. Although these numbers are too small for rigorous statistical analysis, the correlation is striking and clearly indicates that the PDX model may be useful to predict drug sensitivity prospectively. We successfully utilized the HOSC1 model to determine the *in vivo* efficacy of JAK and Src inhibition, demonstrating that our model may be useful for developing novel cancer therapeutics [26].

The biggest limitations of our study were the low engraftment rate and lack of adequate tissue for analysis of all parental tumors. Many resected HNSCC tumors are small, and the residual tissue after standard pathologic analysis is completed is not adequate for research studies. The use of NSG rather than the Nude mouse may improve the engraftment rate and allow for the retention of human fibroblasts and lymphocytes [47-49]. Nonetheless, our study is the most comprehensive analysis and development of the PDX model for HNSCC to date.

## Conclusions

We have demonstrated that developing a PDX model for HNSCC is feasible, although challenging because of the uncertain availability of adequate tumor. The engraftment rate for HNSCC was low, but it may be enhanced by using the NSG mouse, selecting patients with nodal disease and poorly differentiated tumors. Several common themes for PDX tumors were recapitulated by our work: the loss of human stroma; the fidelity of the histology; and the utility for testing therapeutic agents.

## Abbreviations

(HNSCC): Head and neck squamous cell carcinoma; (PDX): Patient-derived xenograft; (NSCLC): Non-small cell lung cancer; (aCGH): Aarray comparative genomic hybridization; (SCLC): Small cell lung cancer; (HOSC): Human oral squamous carcinoma; (PCNA): Proliferating cell nuclear antigen; (NSG): NOD-scid IL2γ^null^; (IHC): Immunohistochemical.

## Competing interests

AW is an employee of Bristol-Myers Squibb which produces dasatinib and BMS911543. The remaining authors declare that they have no competing interests.

## Authors’ contributions

FMJ conceived and designed the project, supervised all the experiments, and wrote the manuscript. SP, BS, and TM helped to design and performed all the animal and bench experiments. CJC and YZ performed the gene expression analysis and prepared the corresponding figures. JM, DB, and MDW assisted with the study design and tissue acquisition, critically reviewed the data, and reviewed the manuscript. Additionally MDW performed all the histologic tissue analyses including interpretation of the IHC and prepared the corresponding figures. AW and ML were involved with the pre-clinical development of BMS-911543 and assisted with the study design and preparation of the manuscript. All authors gave final approval of the manuscript.

## Supplementary Material

Additional file 1: Table S1 Characteristics of patients and implanted tumors. **Table S2.** Histologic characteristics of parent tumors and patient-derived xenografts. **Table S3.** Immunohistochemical analysis with species-specific anti-vimentin antibodies in PDX tumors Click here for file

Additional file 2: Figure S1 Human tumors that engrafted in mice had gene expression profiles distinct from those of human tumors that did not engraft. Click here for file

Additional file 3 **Heterotransplants that grew in mice vs. those that did not.** Heterotransplants that grew in mice/those that did not. Click here for file

Additional file 4 **Heterotransplants that grew in mice vs. those that did not.** Heterotransplants that grew in mice/those that did not. Click here for file

Additional file 5: Figure S2 Human tumors that engrafted in mice had gene expression profiles distinct from those of human tumors that did not engraft. Click here for file

Additional file 6: Figure S3 PDX tumor cells stained for a human-specific epithelial marker. PDXs were stained with anti-keratin 5/6 antibodies that are specific for human keratin using IHC. The stroma did not stain and the intensity of staining for the tumor cells varied widely between the different PDX models. Click here for file

Additional file 7: Figure S4 The growth rates of the PDX models accelerated over time. When the implanted parental tumors grew to 1 cm^3^, it was resected and minced into 2-mm^3^ pieces, which were implanted subcutaneously into mice (F1 generation). The process was repeated to produce subsequent generations. The time for a tumor to reach 1 cm^3^ reflects the growth rate of that tumor. Click here for file

Additional file 8 **Heterotransplant in mice as a group vs. original tumors in humans.** Heterotransplant in mice as a group/original tumors in humans. Click here for file

Additional file 9 **Heterotransplant in mice as a group vs. original tumors in humans.** Heterotransplant in mice as a group/original tumors in humans. Click here for file

Additional file 10: Figure S5 Gene expression of 4 genes in the array demonstrates the same pattern of expression change when confirmed by individual qPCR. We extracted total RNA from HOSC1-F0 and HOSC1-F3 specimens and measured gene expression levels using qPCR for 4 genes that had distinct expression on the array. Two genes that were up regulated on the array (GRIA1, MMP16) were similarly increased in the PDX model as compared to the parental tumor. Likewise, 2 genes that were down regulated in the array (HLA-DRA, LYZ) also demonstrated decreased expression by qPCR. Click here for file
