# Correction: Tumor grafts derived from patients with head and neck squamous carcinoma authentically maintain the molecular and Histologic characteristics of human cancers

**DOI:** 10.1186/1479-5876-12-67

**Published:** 2014-03-13

**Authors:** Shaohua Peng, Chad J Creighton, Yiqun Zhang, Banibrata Sen, Tuhina Mazumdar, Jeffery N Myers, Stephen Y Lai, Adrian Woolfson, Matthew V Lorenzi, Diana Bell, Michelle D Williams, Faye M Johnson

**Affiliations:** 1 Department of Thoracic/Head and Neck Medical Oncology, The University of Texas MD Anderson Cancer Center, Houston, TX, USA; 2 Dan L. Duncan Cancer Center, Baylor College of Medicine, Houston, TX, USA; 3 Department of Head and Neck Surgery, The University of Texas MD Anderson Cancer Center, Houston, TX, USA; 4 Department of Pathology, The University of Texas MD Anderson Cancer Center, Houston, TX, USA; 5 Discovery Oncology, Bristol-Myers Squibb Company, Houston, TX, USA; 6 Department of Medicine, Baylor College of Medicine, Houston, TX, USA; 7 Department of Bioinformatics and Computational Biology, The University of Texas MD Anderson Cancer Center, Houston, TX, USA; 8 The University of Texas Graduate School of Biomedical Sciences, Houston, TX, USA

## Correction

We inadvertently failed to include the complete list of all coauthors for this work [1]. The full list of authors has now been added and the Authors’ contributions and Competing interests section modified.

## Competing interests

AW is an employee of Bristol-Myers Squibb which produces dasatinib and BMS911543. The remaining authors declare that they have no competing interests.

## Authors’ contributions

FMJ conceived and designed the project, supervised all the experiments, and wrote the manuscript. SP, BS, and TM helped to design and performed all the animal and bench experiments. CJC and YZ performed the gene expression analysis and prepared the corresponding figures. JM, DB, MDW, and SYL assisted with the study design and tissue acquisition, critically reviewed the data, and reviewed the manuscript. Additionally MDW performed all the histologic tissue analyses including interpretation of the IHC and prepared the corresponding figures. AW and ML were involved with the pre-clinical development of BMS-911543 and assisted with the study design and preparation of the manuscript. All authors gave final approval of the manuscript.
