# Supplementary material for: Tumor grafts derived from patients with head and neck squamous carcinoma authentically maintain the molecular and histologic characteristics of human cancers
Source: J Transl Med. 2013 Aug 27;11:198. doi: 10.1186/1479-5876-11-198 (PMC3844397; doi:10.1186/1479-5876-11-198)
Supplement: Additional file 1: Table S1 — Characteristics of patients and implanted tumors. Table S2. Histologic characteristics of parent tumors and patient-derived xenografts. Table S3. Immunohistochemical analysis with species-specific anti-vimentin antibodies in PDX tumors [file 1479-5876-11-198-S1.docx]

**Table S1: Characteristics of patients and implanted tumors**

|  |  |  | **Clinical Features** |  |  |  | **Pathologic Features** |  |  |  |
| --- | --- | --- | --- | --- | --- | --- | --- | --- | --- | --- |
| **Case** | **Stage** | **Primary site** | **Time to recurrence (weeks)** | **Time to death (weeks)** | **Follow-up time (weeks)** | **Histology** | **Differentiation** | **ECE** | **HPV** | **Perineural invasion** |
| **HOSC1** | T2N2cM0 | Oral tongue | None | None | 191 | Invasive squamous carcinoma | Moderately differentiated | Present | Not tested | Present |
| **HOSC2** | T2N0M0 | Oral tongue | None | None | 146 | Invasive squamous carcinoma | Moderately differentiated | NA | Not tested | Present |
| **HOSC3** | T4aN0M0 | Maxillary gingiva | 95 | None | 138 | Invasive squamous carcinoma | Moderately differentiated | NA | Not tested | Absent |
| **HOSC4** | Recurrent | Oral tongue | 9 | 17 | 17 | Invasive squamous carcinoma | Moderately differentiated | Absent | Not tested | Present |
| **HOSC5** | T3N0M0 | Oral tongue | None | None | 123 | Invasive squamous carcinoma | Moderately differentiated | NA | Not tested | Absent |
| **HOSC6** | T3N2bM0 | Oral tongue | None | None | 50 | Invasive squamous carcinoma | Moderately differentiated | Absent | Negative | Absent |
| **HOSC7** | T4aN0M0 | Mandibular gingiva | 31 | 57 | 57 | Invasive squamous carcinoma | Moderately differentiated | Absent | Not tested | Absent |
| **HOSC8** | T4aN2b | Oral tongue | 31 | 36 | 36 | Invasive squamous carcinoma | Moderately differentiated | Present | Not tested | Present |
| **HOSC9** | T3N0M0 | Oral tongue | None | None | 121 | Invasive squamous carcinoma | Moderately differentiated | Na | Not tested | Present |
| **HOSC10** | T3N2bM0 | Oral tongue | 21 | 66 | 66 | Invasive squamous carcinoma | Poorly differentiated | Present | Not tested | Present |
| **HOSC11** | T4aN2bM0 | Buccal mucosa | 22 | None | 70 | Invasive squamous carcinoma | Moderately differentiated | Present | Not tested | Absent |
| **HOSC12** | T3N1M0 | Oral tongue | None | None | 19 | Invasive squamous carcinoma | Poorly differentiated | Absent | Negative | Present |
| **HOSC13** | Recurrent | Oral tongue | 23 | >27* | 27 | Invasive squamous carcinoma | Moderately differentiated | Absent | Not tested | Present |
| **HOSC14** | T4aN2bM0 | Buccal mucosa | None | None | 69 | Invasive squamous carcinoma | Moderately differentiated | Absent | Not tested | Absent |
| **HOSC15** | T2N0M0 | Buccal mucosa | None | None | 66 | Invasive squamous carcinoma (verrucoid features) | Well differentiated | Absent | Not tested | Absent |
| **HOSC16*** | Recurrent | Floor of mouth | None | None | 53 | Sarcomatoid squamous carcinoma | Poorly differentiated | Absent | Not tested | Absent |
| **HOSC17** | T3N2bM0 | Oral tongue | 6 | None | 9 | Invasive squamous carcinoma | Poorly differentiated | Present | Not tested | Present |
| **HOSC18** | T2N2bM0 | Oral tongue | 27 | 42 | 42 | Invasive squamous carcinoma | Moderately differentiated | Present | Negative | Absent |
| **HOSC19** | T2N0M0 | Base of tongue | 24 | None | 58 | Basaloid squamous carcinoma | Poorly differentiated | Absent | Positive | Absent |
| **HOSC20** | T4N1M0 | Oral tongue | 22 | NA | 27 | Invasive squamous carcinoma | Poorly differentiated | Absent | Negative | Absent |
| **HOSC21** | T4aN1M0 | Oral tongue | None | None | 55 | Invasive squamous carcinoma | Moderately differentiated | Absent | Negative | Absent |
| **HOSC22** | T3N0M0 | Oral tongue | None | None | 24 | Invasive squamous carcinoma | Moderately differentiated | NA | Not tested | Present |
| **HOSC23** | T2N0M0 | Buccal mucosa | None | None | 50 | Invasive squamous carcinoma | Moderately differentiated | NA | Not tested | Absent |
| **HOSC24** | T4N0M0 | Oral tongue | None | None | 51 | Invasive squamous carcinoma | Moderately differentiated | NA | Not tested | Absent |
| **HOSC25** | Recurrent | Oral tongue | 0 | >27* | 27 | Invasive squamous carcinoma | Well differentiated | Present | Not tested | Present |
| **HOSC26** | T4N0M0 | Mandible | 19 | None | 43 | Invasive squamous carcinoma | Well differentiated | Absent | Not tested | Absent |
| **HOSC27** | T3N0M0 | Retromolar trigone | None | None | 27 | Basaloid squamous carcinoma | Poorly differentiated | Absent | Negative | Absent |
| **HOSC28** | T2N2bM0 | Buccal mucosa | None | None | 20 | Invasive squamous carcinoma | Moderately differentiated | Present | Not tested | Absent |
| **HOSC29** | T4aN2bM0 | Gingiva / mandible | None | None | 7 | Invasive squamous carcinoma | Poorly differentiated | Present | Not tested | Absent |
| **HOSC30** | T1N0M0 | Oral tongue | None | None | 15 | Invasive squamous carcinoma | Moderately differentiated | NA | Not tested | Absent |
| * Exact date of death unknown | | |  |  |  |  |  |  |  |  |
| ECE, extracapsular extension; HPV, human papillomavirus; NA, not applicable  None, no recurrence or death | | |  |  |  |  |  |  |  |  |

**Table S2: Histologic characteristics of parent tumors and patient-derived xenografts**

| **Case** | **Generation** | **Differentiation** | **Stromal Characteristics** | **Perineural Invasion** | **Other Characteristics** |
| --- | --- | --- | --- | --- | --- |
| **HOSC1** | Parent tumor (F0) | Moderate | Desmoplastic with mild inflammatory reaction | Present |  |
|  | F10 | Moderate | No inflammatory reaction | Not evaluable | Areas of necrosis; more homogeneous than F0 |
| **HOSC10** | Parent tumor (F0) | Poor, basaloid | Minimal with an inflammatory reaction | Present |  |
|  | F3 | Poor, basaloid | Minimal | Not evaluable | Small amount of necrosis |
|  | F7 | Poor, basaloid | Minimal | Present | More homogeneous and sheet-like than F0 or F3 |
| **HOSC12** | Parent tumor (F0) | Poor | Abundant stroma and desmoplasia | Present |  |
|  | F1 | Poor, basaloid | Scant stroma | Not evaluable |  |
|  | F3 | Poor, basaloid | Scant stroma | Present | More homogeneous and sheet-like than F0 or F1 |
| **HOSC19** | Parent tumor (F0) | Poor, basaloid nests | Moderate | Absent | Prominent necrosis; areas of tumor sheets |
|  | F1 | Poor, basaloid nests | Moderate | Not evaluable | Central necrosis |
|  | F3 | Poor, basaloid nests | Scant | Not evaluable | More homogeneous and sheet-like than F0 or F1; necrosis present |
| **HOSC21** | Parent tumor (F0) | Moderate | Desmoplastic | Absent | Low amount of inflammation; central necrosis |
|  | F10 | Moderate | Fibrovascular cords | Not evaluable | Scant necrosis |

**Table S3: Immunohistochemical analysis with species-specific anti-vimentin antibodies in PDX tumors**

| **Tumor, Generation** | **Anti-Human Vimentin Staining** | **Anti-Vimentin (Mouse and Human)** |
| --- | --- | --- |
| HOSC1, F3 | Weak tumor cell staining  No stromal or vessel staining | Weak tumor cell staining  Strong stromal staining |
| HOSC10, F3 | No staining in tumor cells  No stromal or vessel staining | No staining in tumor cells  Blood vessels stained positive |
| HOSC12, F1 | No staining in tumor cells  No stromal or vessel staining | About 10% of large cells within sheets of tumor cells stained strongly positive  Weak vessel staining |
| HOSC19, F1 | Weak staining in tumor cells  No stromal or vessel staining | Moderate tumor cell staining  Moderate stromal staining |
| HOSC19, F3 | No staining in tumor cells  No stromal or vessel staining | Weak tumor cell staining  Strong vessel staining |
| HOSC21, F0 | No staining in tumor cells  Strong stromal staining | No tumor cell staining  Strong stromal staining |
| HOSC21 F3 | No staining in tumor cells  No stromal or vessel staining | Weak tumor cell staining  Strong stromal staining |
