# Supplementary figures and images for: Tumor grafts derived from patients with head and neck squamous carcinoma authentically maintain the molecular and histologic characteristics of human cancers
Source: J Transl Med. 2013 Aug 27;11:198. doi: 10.1186/1479-5876-11-198 (PMC3844397; doi:10.1186/1479-5876-11-198)

Supplemental Figure 1A

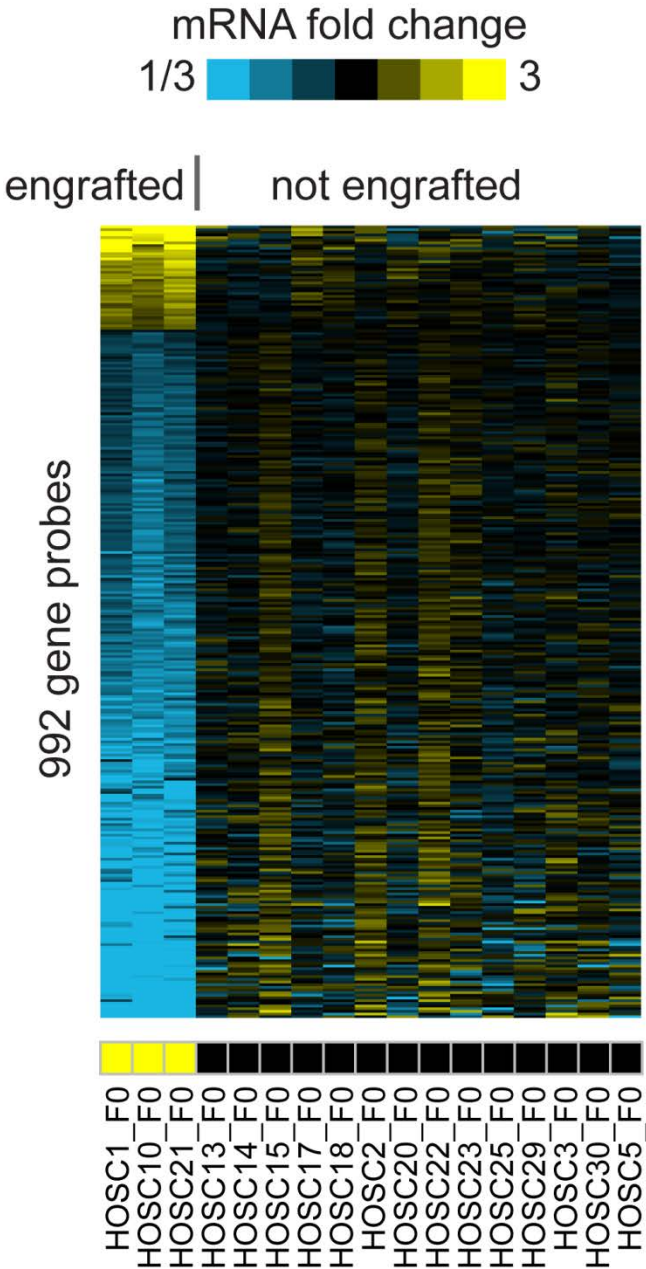

Supplemental Figure 1B

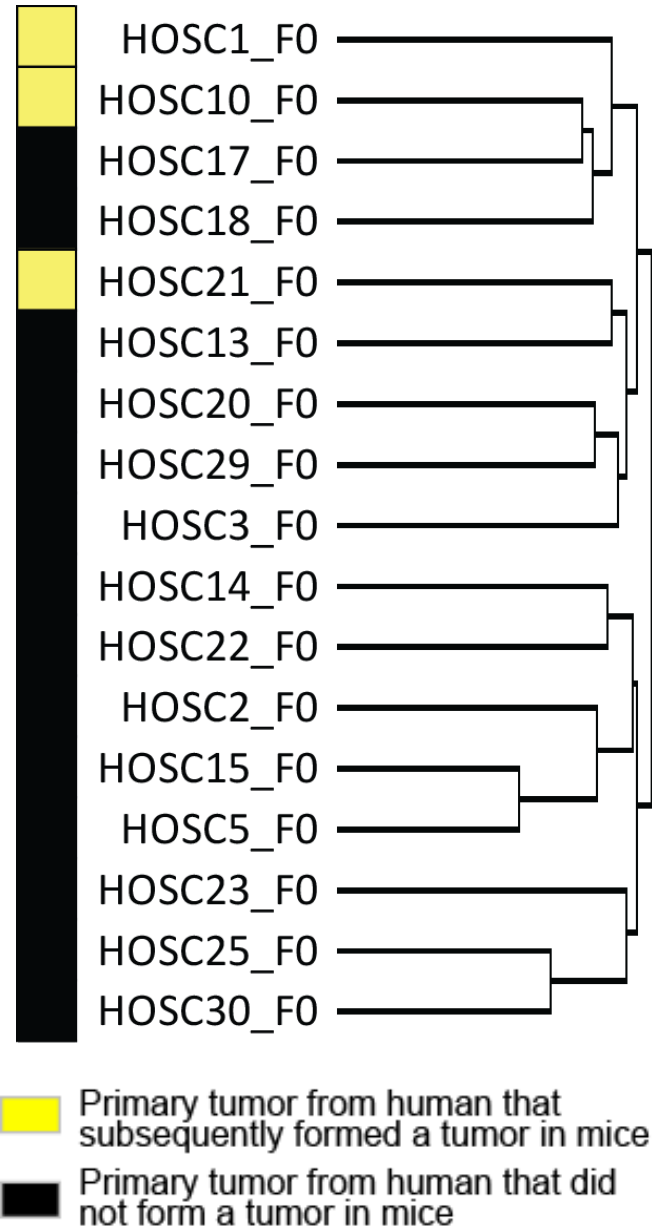

Supplement: Additional file 2: Figure S1 — Human tumors that engrafted in mice had gene expression profiles distinct from those of human tumors that did not engraft. [file 1479-5876-11-198-S2.pdf]

## Supplemental Figure 2

HOSC19 F1

HOSC12 F1

Human-specific  
Antibody

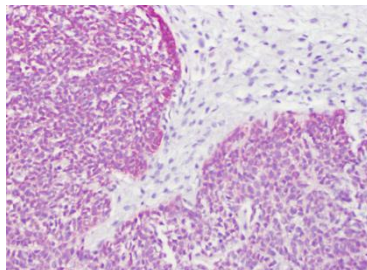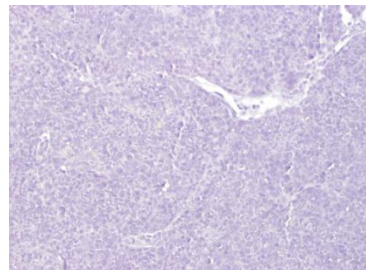

Human- and Mouse-  
specific Antibody

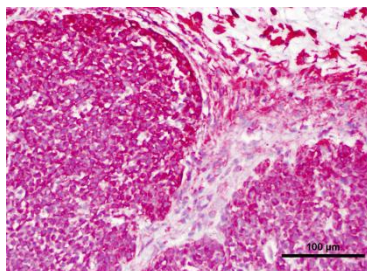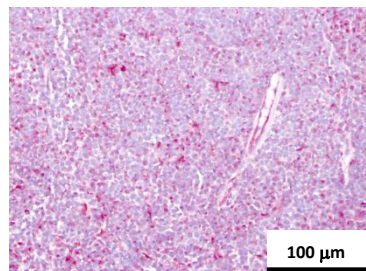

Supplement: Additional file 5: Figure S2 — Human tumors that engrafted in mice had gene expression profiles distinct from those of human tumors that did not engraft. [file 1479-5876-11-198-S5.pdf]

## Supplemental Figure 3

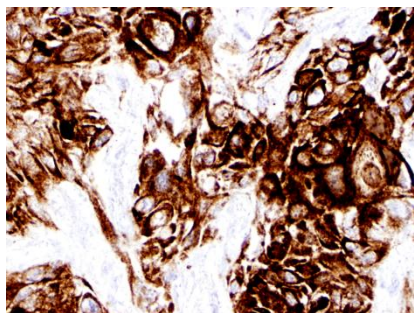

HOSC1 F3 400x

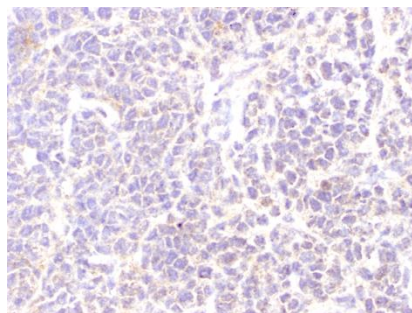

HOSC10 F7 400x

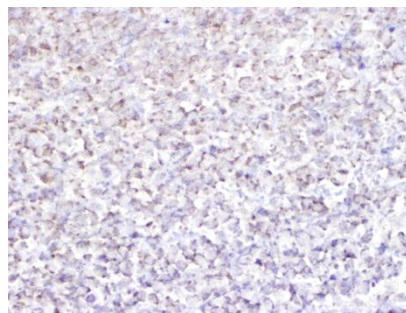

HOSC12 F3 400x

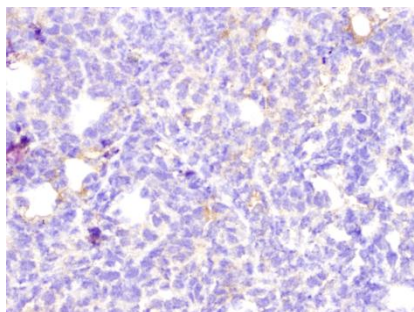

HOSC19 F3 400x

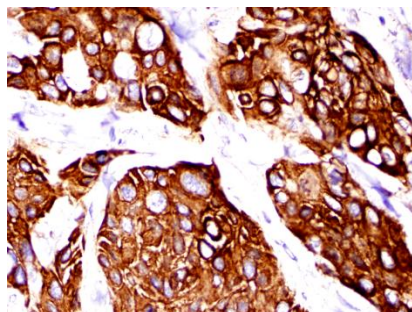

HOSC21 F3 400x

Supplement: Additional file 6: Figure S3 — PDX tumor cells stained for a human-specific epithelial marker. PDXs were stained with anti-keratin 5/6 antibodies that are specific for human keratin using IHC. The stroma did not stain and the intensity of staining for the tumor cells varied widely between the different PDX models. [file 1479-5876-11-198-S6.pdf]

Supplemental Figure 4

**HOSC1**

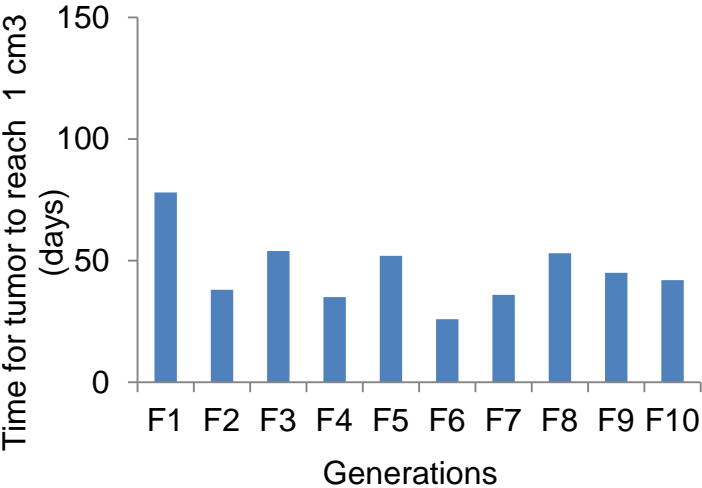

**HOSC10**

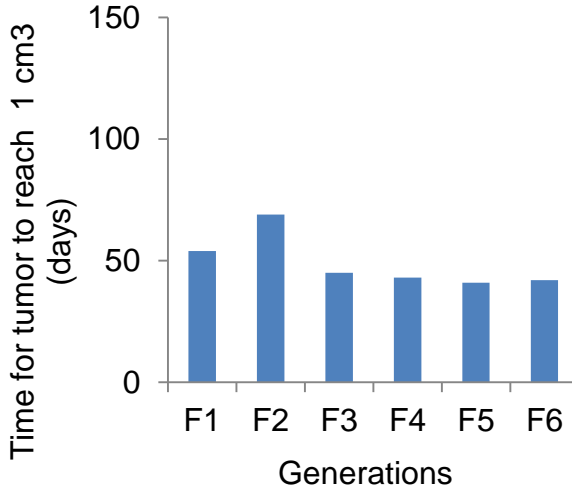

**HOSC12**

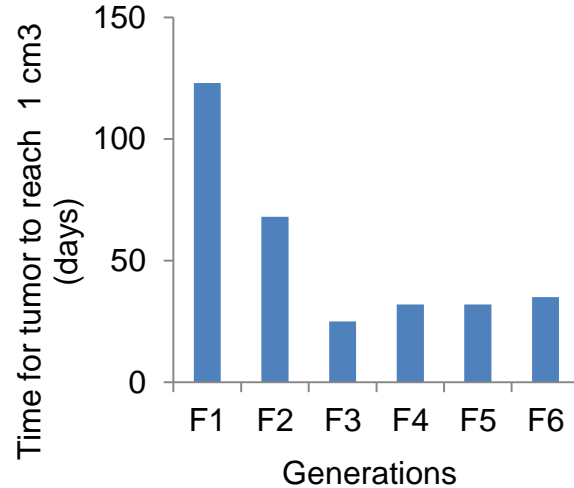

**HOSC19**

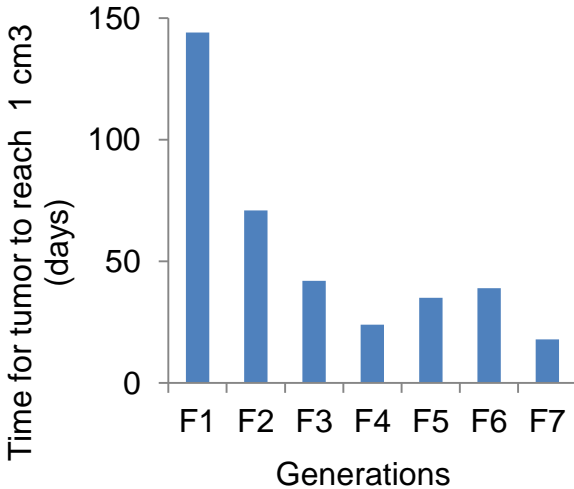

**HOSC21**

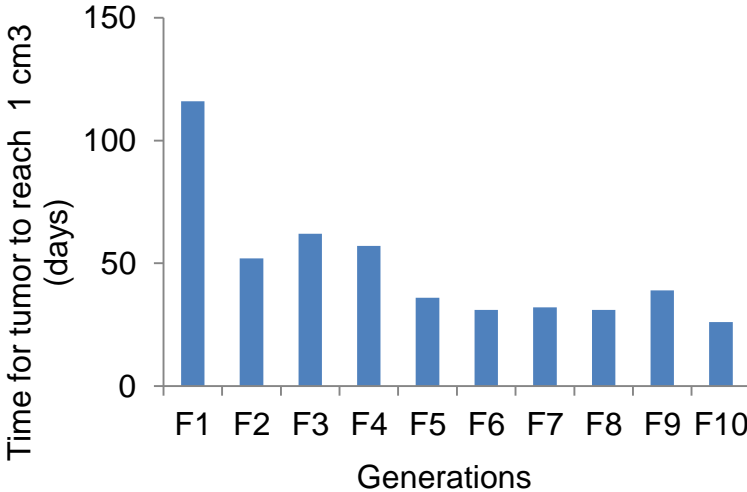

Supplement: Additional file 7: Figure S4 — The growth rates of the PDX models accelerated over time. When the implanted parental tumors grew to 1 cm3, it was resected and minced into 2-mm3 pieces, which were implanted subcutaneously into mice (F1 generation). The process was repeated to produce subsequent generations. The time for a tumor to reach 1 cm3 reflects the growth rate of that tumor. [file 1479-5876-11-198-S7.pdf]

Supplemental Figure 5

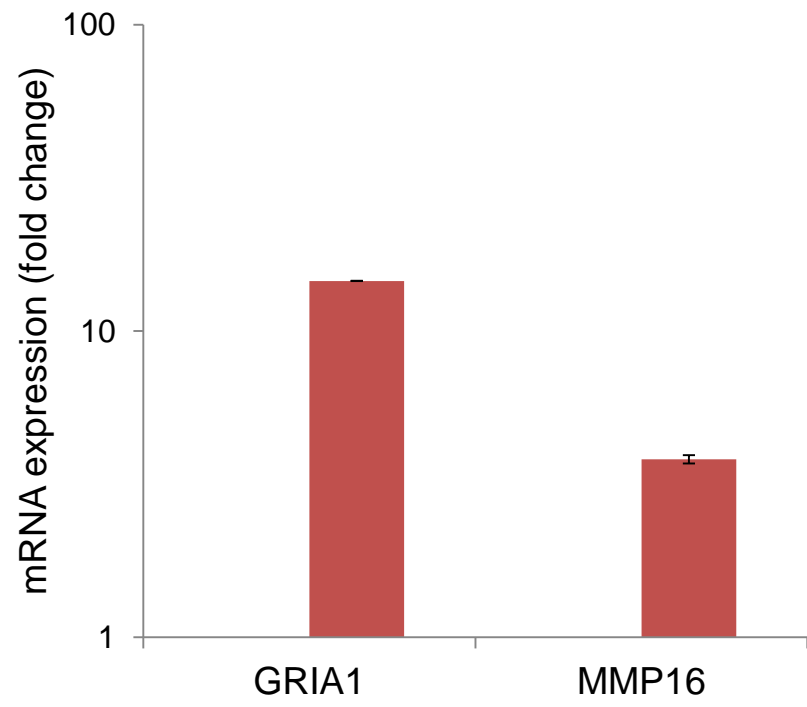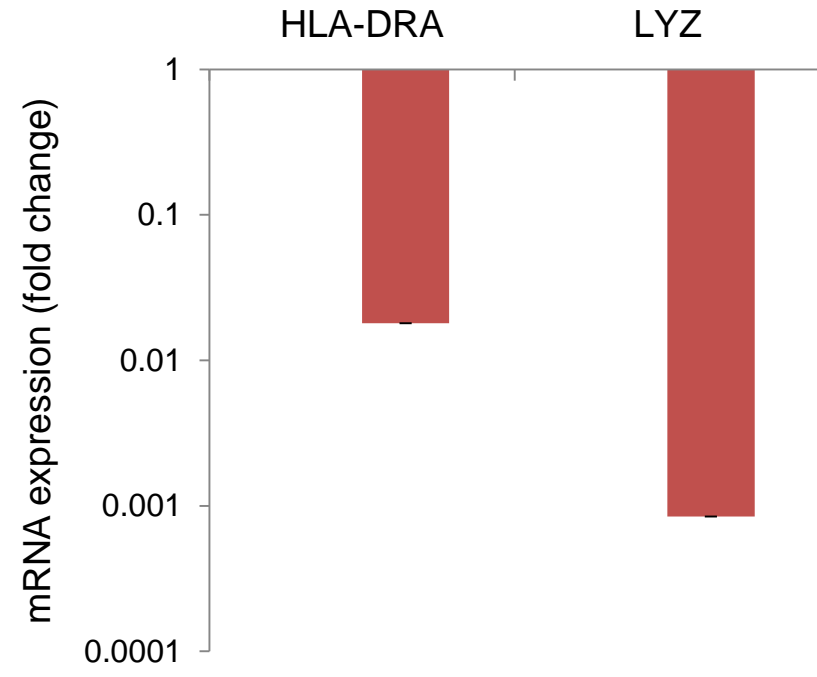

Supplement: Additional file 10: Figure S5 — Gene expression of 4 genes in the array demonstrates the same pattern of expression change when confirmed by individual qPCR. We extracted total RNA from HOSC1-F0 and HOSC1-F3 specimens and measured gene expression levels using qPCR for 4 genes that had distinct expression on the array. Two genes that were up regulated on the array (GRIA1, MMP16) were similarly increased in the PDX model as compared to the parental tumor. Likewise, 2 genes that were down regulated in the array (HLA-DRA, LYZ) also demonstrated decreased expression by qPCR. [file 1479-5876-11-198-S10.pdf]
